# Transient acceleration of autophagic degradation by pharmacological Nrf2 activation is important for retinal pigment epithelium cell survival

**DOI:** 10.1016/j.redox.2018.09.004

**Published:** 2018-09-05

**Authors:** Yuichi Saito, Yoshiki Kuse, Yuki Inoue, Shinsuke Nakamura, Hideaki Hara, Masamitsu Shimazawa

**Affiliations:** Molecular Pharmacology, Department of Biofunctional Evaluation, Gifu Pharmaceutical University, 1-25-4 Daigakunishi, Gifu 501-1196, Japan

**Keywords:** NF-E2-related factor 2, Sequestosome 1, Autophagy, Retinal pigment epithelium, Non-exudative age-related macular degeneration

## Abstract

Non-exudative age-related macular degeneration (AMD) is mainly caused by the accumulation of lipofuscin and drusen on the retinal pigment epithelium (RPE). Both oxidative stress and autophagic dysfunction accelerate the deposition of lipofuscin at the RPE. One of the key regulators in the response against oxidative stress is the NF-E2-Related Factor 2 (Nrf2)-kelch like ECH associated protein 1 (Keap1) axis, which is also closely associated with the autophagy pathway. Nrf2 activation upregulates the expression levels of certain anti-oxidative enzymes [*e.g.* Heme oxygenase-1 (HO-1)], which attenuates oxidative damage. However, until now, the relationship between cytoprotective effects of Nrf2 activation and autophagic degradation remain unclear. To address these questions, we investigated the effects of a novel Nrf2 activator, RS9, on RPE damage. We found that RS9 protected ARPE-19 cells against NaIO_3_-induced oxidative damage, and that the protective effects of RS9 were inhibited by co-treatment with zinc protoporphyrin, an HO-1 inhibitor. Next, we examined the involvement of autophagic degradation in the protective effects of RS9. Co-treatment with RS9 and chloroquine, a lysosomal acidification inhibitor, inhibited the protective effect. Furthermore, western blotting and immunostaining showed that RS9 accelerated autophagy flux and induced transient upregulation of p62 [also known as sequestosome 1 (SQSTM1)]. Co-treatment with chloroquine and RS9 also inhibited the degradation of autophagosomes. Transient upregulation of SQSTM1 by RS9 was unaltered by HO-1 knockdown using siRNA. RS9 and chloroquine had the same actions in light damaged adult zebrafish retina as those *in vitro*. In conclusion, we clarified the relationship between acceleration of the autophagy pathway and the cytoprotective effects of Nrf2 activation in RPE cells and zebrafish retina. These findings indicated that Nrf2 activation could be a promising therapeutic approach for non-exudative AMD by supporting RPE maintenance.

## Graphical abstract

**Figure:**
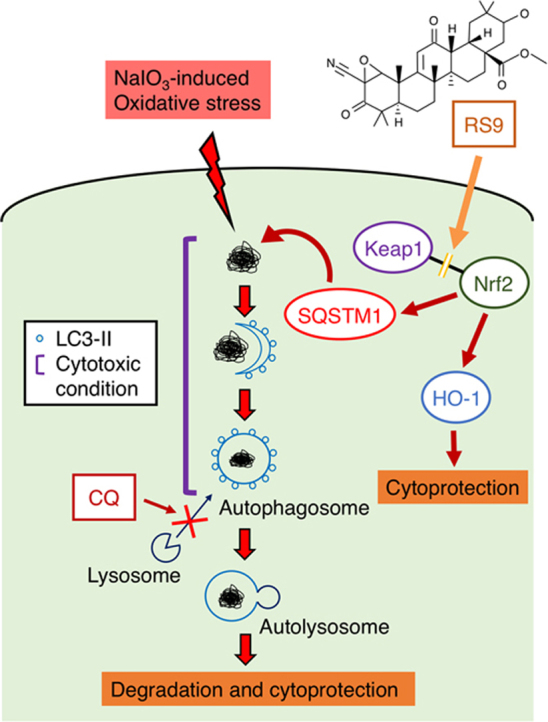

## 1 Introduction

Chronic oxidative stress accelerates the progression of senescence, which then leads to irreversible deposition of cytotoxic waste products. Autophagy, one of the important degradation mechanisms of dysfunctional organelles, is highly required in aged tissues [1]. The activity of the autophagic degradation is related to several age-dependent pathogeneses, such as tau deposition in patients with Alzheimer's disease [2], [3], and lipofuscin accumulation in patients with age-dependent macular degeneration (AMD) [4]. To analyze these age-related pathologies in detail, it is essential to understand the relationship between the cell damage induced by oxidative stress and the autophagic degradation mechanism. However, until now, detailed and comprehensive findings are insufficient, particularly in ophthalmology.

Non-exudative AMD is the leading cause of irreversible vision loss in elderly patients. Effective therapeutics for non-exudative AMD have not yet been developed [5], [6]. The pathogenesis of this disease is somewhat unclear; however, one of the important changes is the accumulation of lipofuscin on the retinal pigment epithelium (RPE). Lipofuscin comprises lysosomal insoluble pigment granules that persist after lysosomal digestion [7]. Lipofuscin formation is closely associated with dysregulated autophagy. Indeed, an autophagy inducer, rapamycin, decreased lipofuscin accumulation in RPE cells [4]. Rapamycin also attenuated the cell damage induced by A2E, a lipofuscin fluorophore [8]. Taken together, the acceleration of autophagy flux may be a potential therapeutic target for non-exudative AMD.

The nuclear transcription factor Nrf2 [nuclear factor erythroid 2 (NF-E2)-related factor 2] is a key transcriptional regulator of defense responses to oxidative stress. Keap1 (kelch-like ECH-associated protein 1) is a suppresser of Nrf2 activity [9]. Nrf2 is activated when cells are exposed to oxidative stress. To date, a wide variety of Nrf2 activators have been reported [10], [11], [12], [13]. Among them, a highly effective novel triterpenoid, RS9, was recently isolated as an Nrf2-specific activator [12], [14]. Moreover, RS9 induced Nrf2-dependent proteins, such as Heme oxygenase-1 (HO-1) and NADPH: quinone oxidoreductase-1 (NQO-1), at lower concentrations compared with Bardoxolone methyl, a previously reported Nrf2 activating triterpenoid [14], [15]. RS9 showed protective effects on light-induced photoreceptor cell death *in vitro* and *in vivo* [16]. Moreover, the Nrf2 activating triterpenoid RTA408 also showed protective effects on the RPE against H_2_O_2_-induced oxidative cell damage [13].

Several studies have demonstrated that the Nrf2-Keap1 axis is closely related to autophagic degradation [17], [18], [19], [20]. However, the involvement of autophagic degradation in these protective effects remains unclear. To elucidate this mechanism, we used the NaIO_3_-induced RPE cell death model. NaIO_3_ is an oxidant that causes oxidative cell damage to the RPE, and is frequently used to induce RPE cell damage *via* oxidative stress [21], [22], [23], [24]. Previous studies indicated that NaIO_3_ increases the levels of abnormal cytotoxic unfolded proteins [25], [26]. Thus, NaIO_3_ is used as a model of non-exudative AMD [27]. Autophagy plays an essential role in the clearance of aggregated toxic proteins and degradation of damaged organelles [28]. Therefore, using the *in vitro* NaIO_3_-induced RPE cell damage model, we investigated the relationships between the cytoprotective effects of Nrf2 activation and autophagic degradation under oxidative stress.

## 2 Material and methods

### 2.1 Cell culture

The RPE cell line (ARPE-19) was purchased from the American Type Culture Collection (Manassas, VA, USA). The cells were maintained in Dulbecco's modified Eagle's medium (DMEM)/F-12 (Wako, Osaka, Japan) containing 10% fetal bovine serum (FBS), 100 U/mL penicillin, and 100 µg/mL streptomycin. Cultures were maintained at 37 °C under a humidified atmosphere of 95% air and 5% CO_2_. The cells were passaged using trypsinization every 4 or 5 days.

### 2.2 NaIO_3_-induced cell death assay

The ARPE-19 cells were seeded at a density of 1.5 × 10^4^ cells per well into 96-well plates, and then incubated for 4 days. NaIO_3_ (Sigma-Aldrich, St. Louis, MO, USA) was diluted in phosphate-buffered saline (PBS), and used to treat the cells at a final concentration of 10 mM [29]. The medium was changed to FBS free DMEM/F-12 for 1 h before the start of NaIO_3_ treatment. In all the *in vitro* experiments, the cells were evaluated using the subsequent assay procedures at 24 h after treatment.

### 2.3 Reagents

RS9 was a kindly gift from Daiichi Sankyo Co., Ltd. (Tokyo, Japan) and treatment started 6 h before NaIO_3_ treatment. N-acetyl cysteine (NAC) (Wako) and chloroquine (Wako) were used to treat the cells at the same time as RS9. Zinc protoporphyrin (ZnPP) (Frontier Scientific Inc., Logan, Ut, USA) was used to treat 1 h before NaIO_3_ treatment.

### 2.4 Cell viability assay

We examined the change in the fluorescence intensity after the cellular mitochondrial reduction of WST-8 to formazan. Cell viability was measured by culturing the cells in a culture medium containing 10% WST-8 (Cell Counting Kit-8; Dojin Kagaku, Kumamoto, Japan) for 3 h at 37 °C and then by scanning the absorbance at 450 nm using a microplate reader (GloMax-Multi Detection System; Promega, Madison, WI, USA).

### 2.5 Cell death analysis

The cell death rate was calculated by double staining with two fluorescent dyes: Hoechst 33342 (Thermo Fisher Scientific, Waltham, MA, USA) and propidium iodide (PI; Thermo Fisher Scientific). Hoechst 33342 stains the nuclei of all cells, whereas PI stains only dead cells. At the end of the culture period, Hoechst 33342 and PI were added to the culture medium for 15 min at final concentrations of 8.1 mM and 1.5 mM, respectively. Images were collected using a Lionheart FX automated microscope (BioTek, Winooski, VT, USA). The total number of cells was counted automatically using the Gen5 software (BioTek) and the percentage of PI-positive cells was calculated.

### 2.6 Mitochondrial membrane potential assay

The mitochondrial membrane potential was evaluated using a JC-1 Mitochondrial Membrane Potential Assay Kit (Thermo Fisher Scientific) according to the manufacturer's protocol. The representative images were captured using a BZ-X700 all-in-one fluorescence microscope (Keyence, Osaka, Japan), which detects healthy cells with mainly JC-1 aggregates (excitation/emission = 540/605 nm) and apoptotic or unhealthy cells with mainly JC-1 monomers (excitation/emission = 480/510 nm). Merged cells were considered to be pre-apoptotic (the early or middle state of the transition to cell death). For the quantitative analysis, the fluorescence intensity of JC-1 aggregates and JC-1 monomers were evaluated using a microplate reader (GloMax-Multi Detection System; Promega). The relative intensity (green/red) was corrected by the cell number, which was obtained by counting the Hoechst 33342-positive cells. The numbers of Hoechst 33342-positive cells were counted automatically using the BZ-X700 cell analyzer system.

### 2.7 Western blotting analysis

The ARPE-19 cells were cultured in 24-well plates. At the evaluation time points, cells were washed with PBS, lysed using radioimmunoprecipitation assay (RIPA) buffer (Sigma-Aldrich) containing 1% protease inhibitor cocktail and 1% of phosphatase inhibitor cocktails 2 and 3 (Sigma-Aldrich), and harvested. The lysates were centrifuged at 12,000 rpm for 20 min at 4 °C. Protein concentrations were measured using a BCA protein assay kit (Thermo Fisher Scientific), with bovine serum albumin (BSA) as a standard. Lysates were mixed with sample buffer containing 10% 2-mercaptoethanol, and subjected to 10% sodium dodecyl sulfate-polyacrylamide gel electrophoresis (SDS-PAGE). The separated proteins were then transferred onto a polyvinylidene difluoride membrane (Immunobilon-P, Merck KGaA, Darmstadt, Germany). The membranes were incubated with the following primary antibodies: anti-HO-1 rabbit polyclonal (1:1000, Merck KGaA), anti-microtubule-associated protein 1 light chain 3 (LC3) rabbit monoclonal (1:1000, Cell signaling technology (CST), Beverly, MA, USA), anti-sequestosome 1 (SQSTM1)/p62 mouse monoclonal (1:500, GeneTex, Irvine, CA, USA), anti-glyceraldehyde-3-phosphate dehydrogenase (GAPDH) rabbit monoclonal (1:1000, CST). The membrane was further incubated with the secondary antibodies including horseradish peroxidase (HRP)-conjugated goat anti-rabbit IgG (1:2000, Thermo Fisher Scientific), and HRP-conjugated goat anti-mouse IgG (1:2000, Thelrmo Fisher Scientific). The immunoreactive protein bands were visualized using Immunostar-LD (Wako) and imaged using an LAS-4000 luminescent image analyzer (Fuji Film Co., Ltd., Tokyo, Japan). GAPDH was used as the loading control.

### 2.8 Immunostaining

The ARPE-19 cells were cultured in 8-well chamber slides (SCS-N08, Matsunami Glass Ind., Ltd., Osaka, Japan). At the evaluation time points, the cells were washed with PBS, then fixed with 4% paraformaldehyde (Wako) for 15 min, blocked with 3% goat serum for 30 min, and then incubated overnight at 4 °C with the anti-LC3 rabbit monoclonal antibody (1:200, CST). After washing, the cells were incubated for 1 h with the secondary antibody (Alexa Fluor 488-conjugated goat anti-rabbit IgG, Thermo Fisher Scientific). The cells were washed again and then incubated overnight at 4 °C with anti-SQSTM1/p62 mouse monoclonal antibody (1:500, GeneTex). After washing, the cells were incubated for 1 h with the secondary antibody (Alexa Fluor 546-conjugated goat anti-mouse IgG, Thermo Fisher Scientific). The cells were then washed again and counter-stained with Hoechst 33342 (1:1000, Thermo Fisher Scientific). Images were acquired using a confocal fluorescence microscope (FLUOVIEW FV10i; Olympus, Tokyo, Japan). Autophagosomes and SQSTM1-positive speckles were detected using image-processing software, Image-J (developed by the National Institutes of Health).

### 2.9 RNA interference

Stealth RNAi™ siRNA Duplex Oligo ribonucleotides [Hmox1 RNAi (Thermo Fisher Scientific) and Stealth RNAi™ siRNA Negative Controls (Thermo Fisher Scientific)] for HO-1 and negative control small interfering RNAs (siRNA) were used for RNA interference. The sense and antisense strands of HO-1 siRNA were as follows:

sequence #1, 5′-CAGCUCUAUCGUGCUCGAAUGAACA-3′ (sense)

and 5′-UGUUCAUUCGAGCACGAUAGAGCUG-3′ (antisense);

sequence #2, 5′-GCUUUAAGCUGGUGAUGGCUUCCUU-3′ (sense)

and 5′-AAGGAAGCCAUCACCAGCUUAAAGC-3′ (antisense);

sequence #3, 5′-GGCAGUGGGAAUUUAUGCCAUGUAA-3′ (sense)

and 5′-UUACAUGGCAUAAAUUCCCACUGCC-3′ (antisense).

### 2.10 In vitro siRNA transfection

To assess the effect of HO-1 siRNA knockdown, The ARPE-19 cells were cultured in 24-well plates containing the standard cultured medium at 37 °C for 4 days. Then the medium was changed to antibiotic-free standard culture medium and transfected with 10 nM of siRNA using Lipofectamine™ RNAiMAX Reagent (Thermo Fisher Scientific) and Opti-MEN (Thermo Fisher Scientific) according to the manufacturer's protocol. After 24 h of exposure, the medium were replaced by DMEM containing 10% FBS and RS9 was treated. Five hours after the treatment, the medium were replaced by DMEM without FBS and RS9 was treated, and then incubated for 24 h.

### 2.11 Breeding zebrafish

Adult pigmented wild-type zebrafish (WT) were obtained from RIKEN-wako. Zebrafish were maintained in cycles of 14 h of light: 10 h of dark at 28.5 °C [30]. Adult fish (4 months old) were used for all the experiments. All experiments were performed in accordance with the Association for Research in Vision and Ophthalmology (ARVO) Statement for the Use of Animals in Ophthalmic and Vision Research and approval by the Gifu Pharmaceutical University Committee on the Use and Care of Animals.

### 2.12 Light induced-retinal degeneration assay and histochemistry

The experiment was conducted in accordance with a previously reported procedure [31]. RS9 was diluted in PLA-0020, a poly-lactide (20,000 Da; Wako), at a ratio of 1:9 of RS9 to PLA-0020. This solution was made so the RS9 would be released slowly [14], [16]. Six hours before light irradiation, 1 µL of 3 mM RS9 was injected into the vitreous humor by inserting a 34-gauge needle through the corneal-scleral junction under anesthesia using 0.1% phenoxyethanol solution. We used intravitreal injection of the equivalent concentration of PLA-0020 as a vehicle control. Chloroquine treatment at 300 µM also injected intravitreally at the same time.

### 2.13 Statistical analysis

Data are presented as means ± standard error of the mean (SEM). The statistical analyses were performed using the SPSS statistical software package (IBM, Armonk, NY, USA). Statistical comparisons were performed using one-way analysis of variance (ANOVA) with Tukey's test or Student's *t*-test. A value of P < 0.05 was considered statistically significant.

## 3 Results

### 3.1 RS9 suppressed the ARPE-19 cell damage induced by NaIO_3_ treatment *via* the HO-1 pathway

**Fig. 1:**
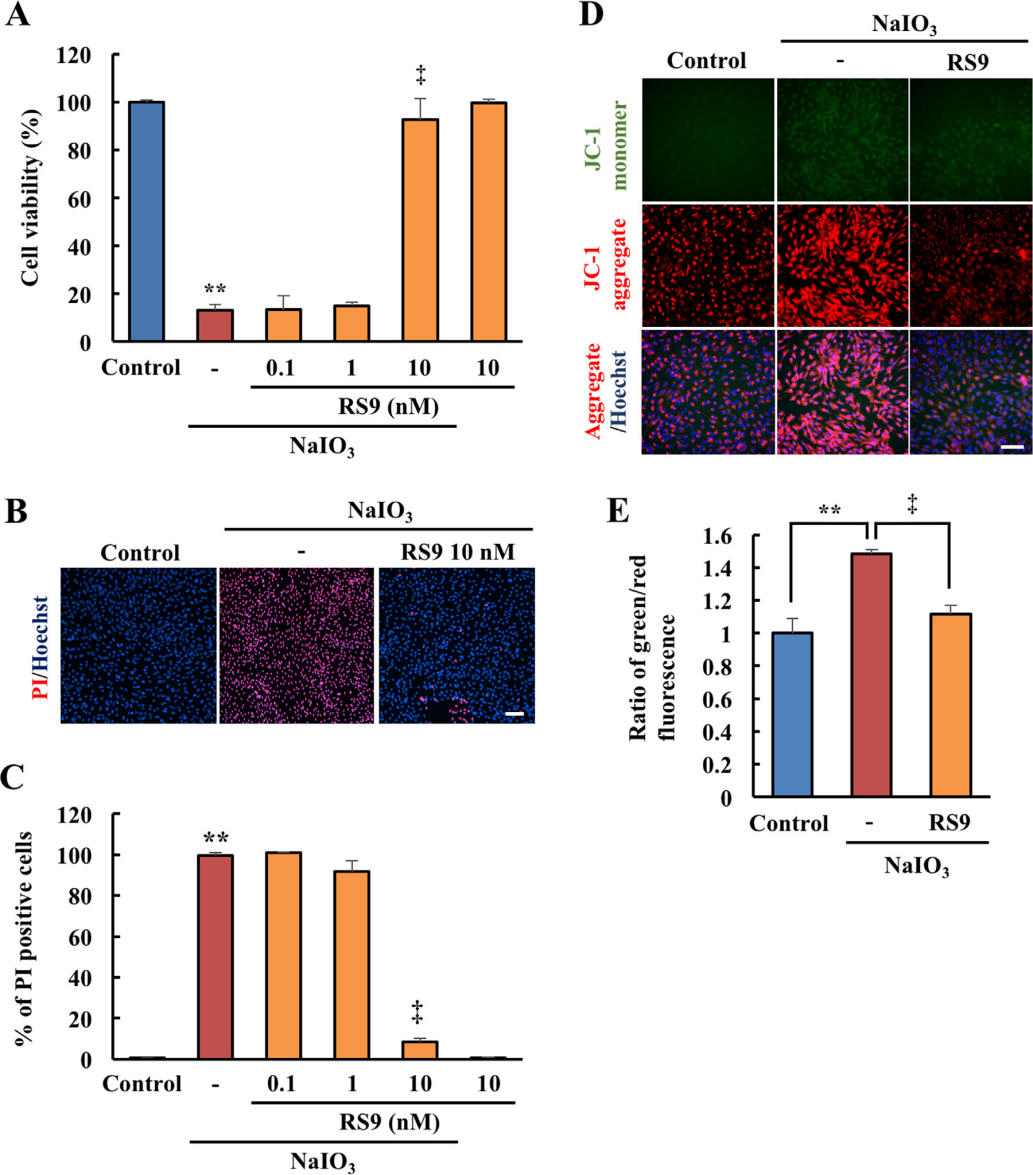
**RS9 showed protective effects on ARPE-19 cells against NaIO**_**3**_**-induced oxidative stress.** (A) Cell viability was evaluated using the Cell Counting Kit (CCK)−8 assay. (B-C) Cell death rates were evaluated by propidium iodide (PI) (red) /Hoechst 33342 (blue) double staining. (B) Representative images. (C) Quantitative data of the percentage of PI positive cells. (D-E) The ratio of mitochondria that had an impaired membrane potential (JC-1 monomer: green) to healthy mitochondria (JC-1 aggregate: red) was evaluated using the JC-1 assay. (D) Representative images. (E) Quantitative data for the ratio of monomers to aggregates. Scale bar = 100 µm. In all experiments, NaIO_3_ was used at 10 mM final concentration. Scale bar = 100 µm. Mean ± SEM (n = 6), ^**^P < 0.01 *vs*. control, and ^‡^P < 0.01 *vs*. vehicle (Tukey's test).

**Fig. 2:**
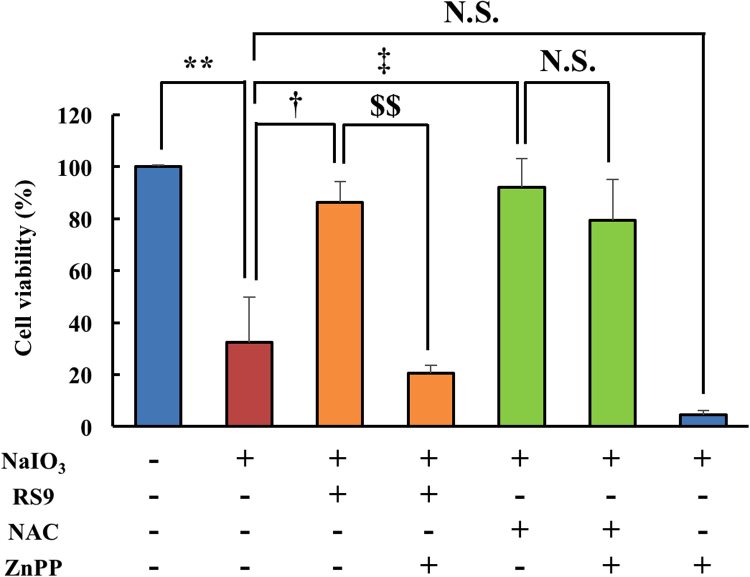
**ZnPP abolished the protective effects of RS9, but did not abolish the protective effects of NAC.** Cell viability was evaluated using the CCK-8 assay. NaIO_3_, RS9, N-acetyl cysteine (NAC), zinc protoporphyrin (ZnPP) were used at 10 mM, 10 nM, 1 mM, and 3 μM final concentration, respectively. Mean ± SEM (n = 6), ^**^P < 0.01 *vs*. control, ^‡^P < 0.01 *vs*. NaIO_3_ (+), and ^$$^P < 0.01 *vs.* NaIO_3_ (+), RS9 (+) (Tukey's test). N.S.: not significant.

We firstly verified the effects of RS9 on the RPE cell damage induced by NaIO_3_ treatment. WST-8 assays and cell death analysis showed that RS9 had concentration-dependently protective effects on ARPE-19 cells under NaIO_3_-treatment (Fig. 1 A-C). Moreover, we investigated the mitochondria membrane potential, which is disrupted during early apoptosis. The JC-1 assay showed that NaIO_3_ treatment increased the level of the JC-1 monomer (green), which marks mitochondria with an impaired membrane potential. RS9 at 10 nM significantly decreased the ratio of JC-1 monomer to JC-1 aggregates, which indicates normal mitochondria (Fig. 1 D-E). Then, we studied the relationships between the protective effects of RS9 and the Nrf2-Keap1 axis. HO-1 is an antioxidant protein mainly induced after activation of Nrf2. To determine the involvement of HO-1, we used the HO-1 inhibitor, ZnPP [32]. A WST-8 assay showed that ZnPP treatment completely abolished the protective effects of RS9. In contrast to RS9, the protective effects of NAC were not canceled by ZnPP treatment (Fig. 2). Taken together, these results indicated that RS9 protected RPE cells from NaIO_3_-induced oxidative stress *via* HO-1 induction.

### 3.2 RS9 protected ARPE-19 cells *via* the autophagic pathway

**Fig. 3:**
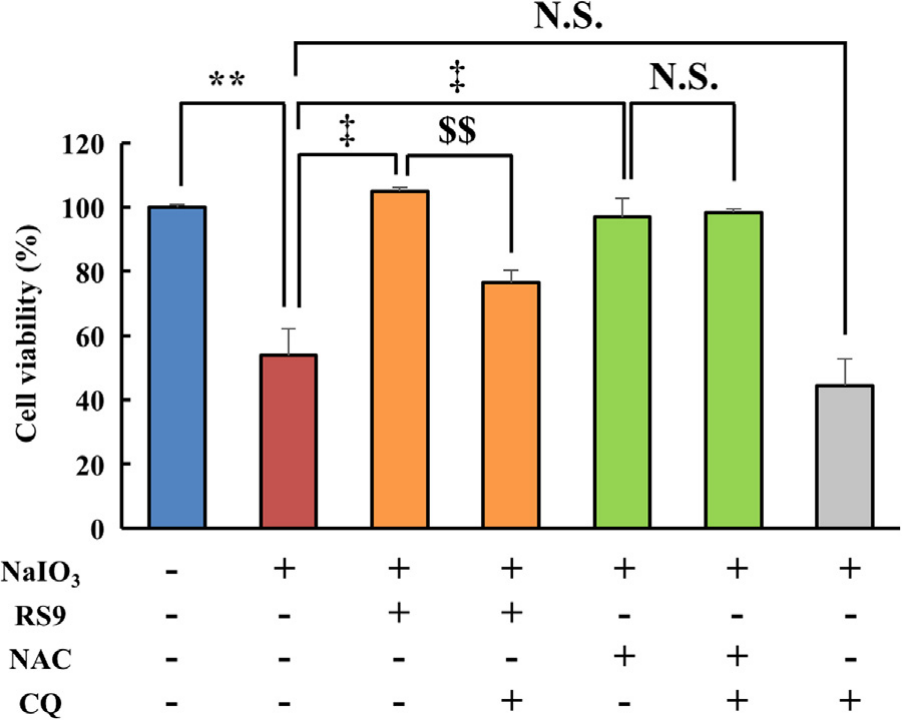
**Chloroquine abolished the protective effects of RS9, but did not abolish the protective effects of NAC.** Cell viability was evaluated by the CCK-8 assay. NaIO_3_, RS9, NAC, chloroquine (CQ) were used at 10 mM, 10 nM, 1 mM, and 30 μM final concentration, respectively. Mean ± SEM (n = 6–12), ^**^P < 0.01 *vs*. control, ^‡^P < 0.01 *vs*. NaIO_3_ (+), and ^$$^P < 0.01 *vs*. NaIO_3_ (+), RS9 (+) (Tukey's test). N.S.: not significant.

We next tried to elucidate the contribution of autophagic degradation to the protective effects of RS9. Chloroquine is widely known as a lysosomal acidification inhibitor that inhibits degradation of autophagosome. Hence, we used this compound as an autophagy flux inhibitor [33]. Cell viability assays showed that chloroquine treatment considerably suppressed the protective effects of RS9. In contrast to RS9, the protective effects of NAC were not canceled by chloroquine treatment (Fig. 3). Based on this result, we hypothesized that the autophagic pathway might regulate the protective effects of RS9.

### 3.3 RS9 accelerated autophagy flux *via* transient upregulation of p62/SQSTM1

**Fig. 4:**
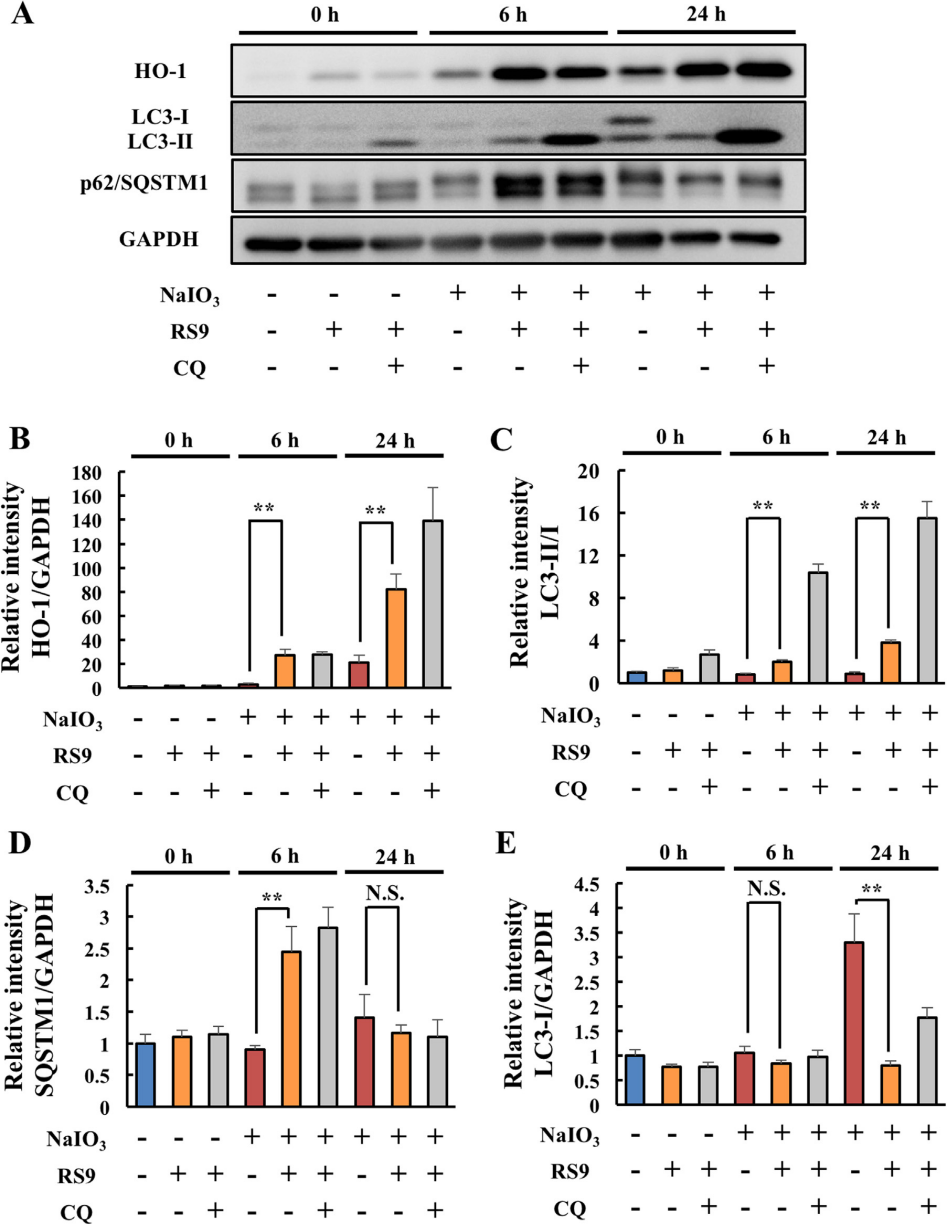
**RS9 upregulated the expression levels of HO-1, LC3-II, and SQSTM1.** (A) Representative results of the time course analysis by western blotting. (B-E) The results of quantitative analysis of the intensity of each immunoreactive protein band. (B) HO-1 expression levels normalized to those of GAPDH. (C) LC3-II levels compared with those of LC3-I. (D) SQSTM1 levels normalized to those of GAPDH. (E) LC3-I levels normalized to those of GAPDH. NaIO_3_, RS9, CQ were used at 10 mM, 10 nM, and 30 μM final concentration, respectively. Mean ± SEM (n = 4), ^**^P < 0.01, (Student's *t*-test). N.S.: not significant.

To gain a further understanding, we evaluated the levels of autophagy-related proteins using western blotting analysis just before the NaIO_3_ treatment (0 h), 6 or 24 h after the NaIO_3_ treatment. HO-1 levels were significantly upregulated by RS9 treatment compared with those induced by NaIO_3_ alone at both 6 and 24 h after NaIO_3_ treatment (Fig. 4 A-B). The ratio of LC3-II to LC3-I was also upregulated by RS9 treatment at both 6 and 24 h after NaIO_3_ treatment. Chloroquine co-treatment further upregulated the levels of these proteins (Fig. 4 A, C). Notably, the autophagy substrate p62/SQSTM1 was transiently upregulated by RS9 treatment only at 6 h after NaIO_3_ treatment (Fig. 4 A, D). The levels of LC3-I were upregulated in the NaIO_3_ treatment group only at 24 h after treatment. By contrast, RS9 treatment maintained LC3-I at normal levels (Fig. 4 A, E). These results indicated that RS9 accelerated autophagic flux *via* transient induction of SQSTM1.

### 3.4 RS9 transiently increased autophagosomes and SQSTM1-positive speckles

**Fig. 5:**
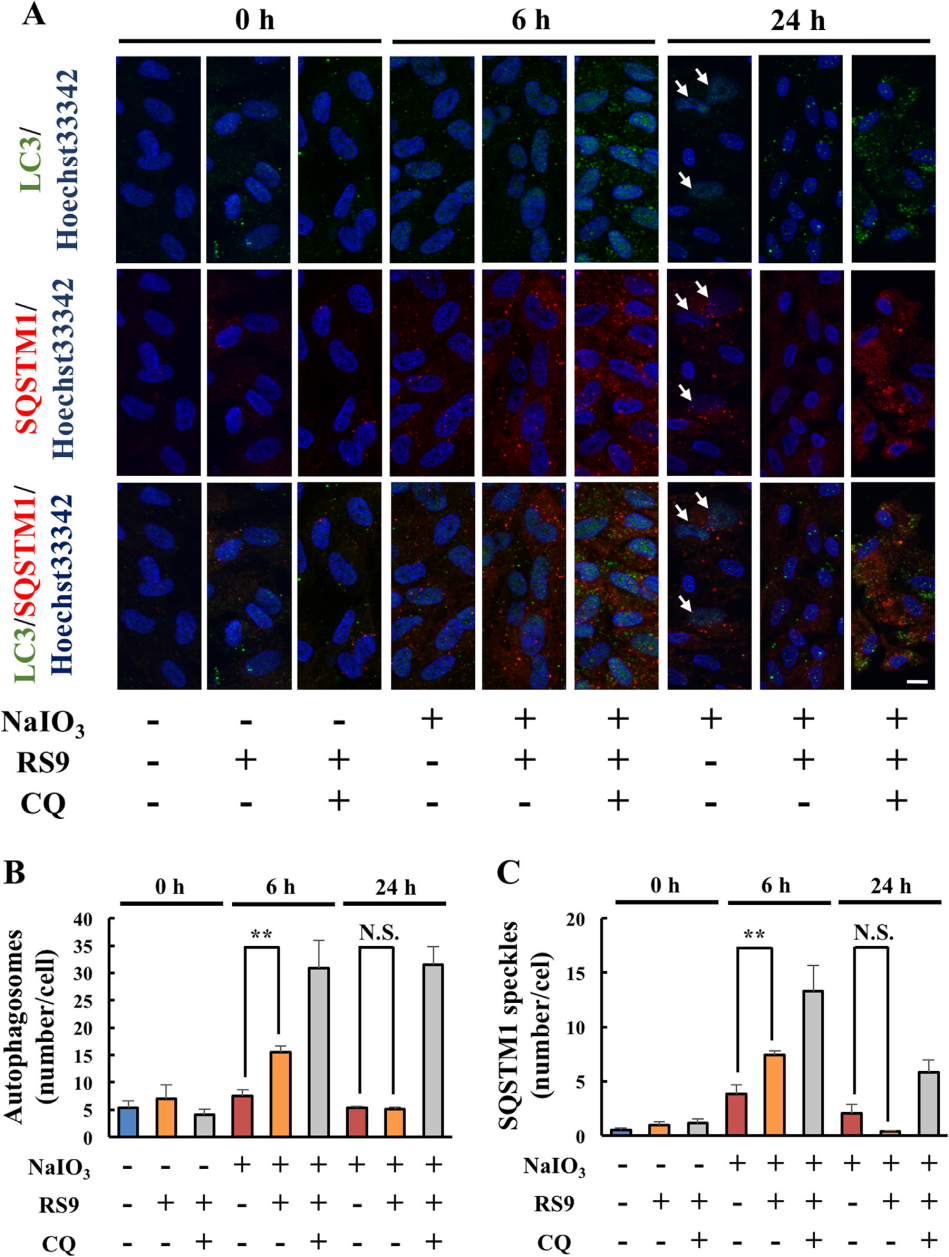
**RS9 transiently accelerated autophagy flux through SQSTM1 induction.** (A) Representative images of LC3 and SQSTM1 immunostaining (LC3: green, SQSTM1: red, Hoechst 33342: blue). White arrows indicate the vacuolated and swollen formed nuclei. (B) Quantitative analysis of the number of LC3 positive autophagosomes. (C) Quantitative analysis of the number of SQSTM1-positive speckles. Scale bar = 10 µm. Mean ± SEM (n = 4), ^**^P < 0.01, (Student's *t*-test). N.S.: not significant.

We also evaluated the changes in LC3 and SQSTM1 levels using immunostaining. Immunostaining showed that the number of LC3 puncta in the cytoplasm, which represent autophagosomes, was transiently increased by RS9 treatment compared with NaIO_3_ only treatment at 6 h after treatment. Chloroquine co-treatment, which should inhibit the degradation of autophagosomes, consequently and drastically increased the cellular accumulation of LC3-positive autophagosomes at both 6 and 24 h after NaIO_3_ treatment (Fig. 5 A-B). Under RS9 treatment, SQSTM1-positive speckles were transiently increased at 6 h after NaIO_3_ treatment, similar to the results of the western blotting analysis (Fig. 5 A, C). Moreover, at 24 h after NaIO_3_ treatment, we detected some of nuclei in the NaIO_3_ treatment alone group that not only showed abnormal Hoechst 33342 staining and were vacuolated and swollen, but also showed high intensity of LC3 and SQSTM1 staining (Fig. 5 A, white arrows). These nuclei were not detected in the RS9 alone groups and the RS9 and chloroquine co-treatment group. These results confirmed that RS9 transiently activates autophagy flux and is probably related to RPE cell survival.

### 3.5 Transient induction of SQSTM1 by RS9 was unaltered after HO-1 knockdown

**Fig. 6:**
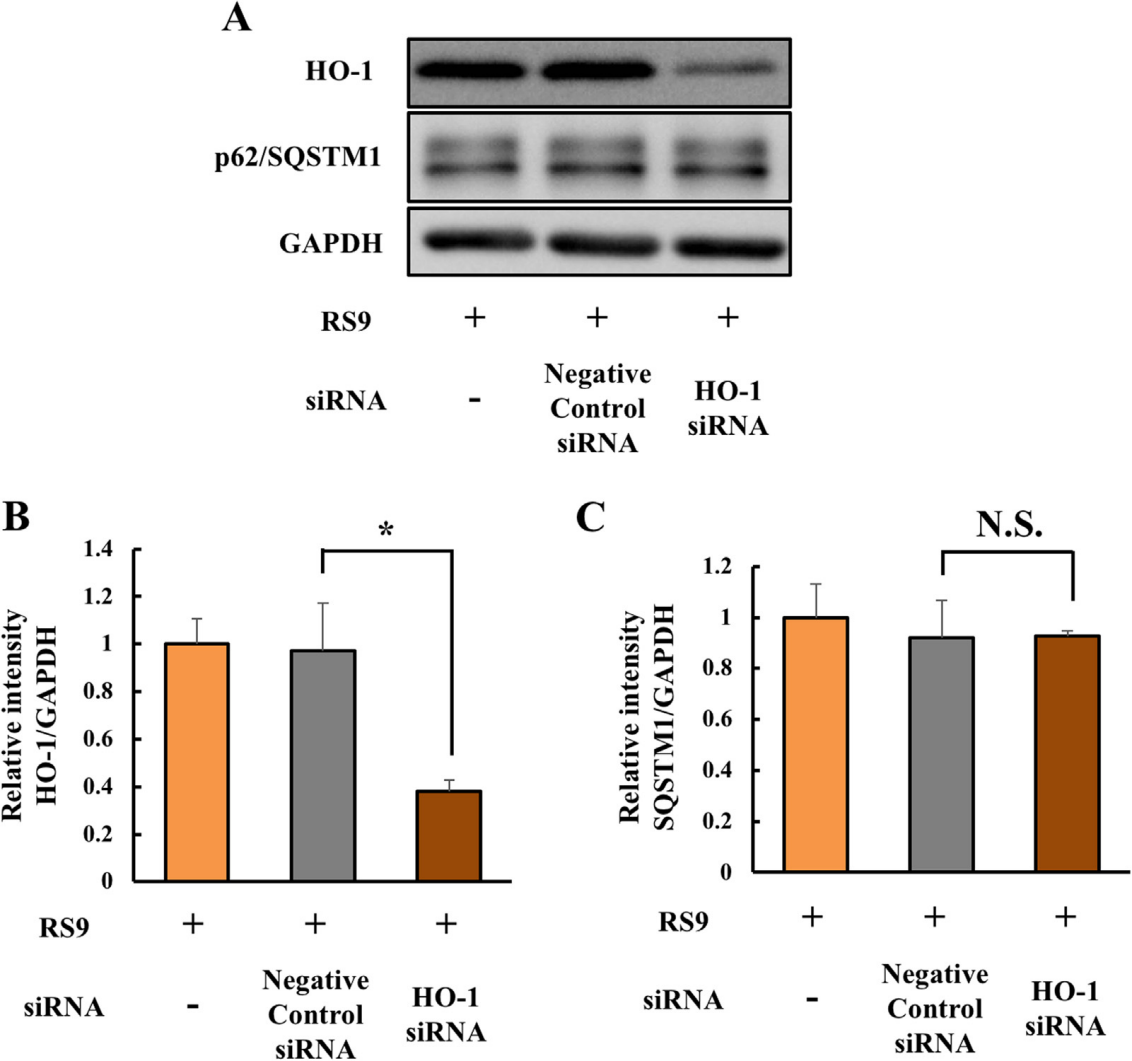
**SQSTM1 induction by RS9 was unaltered by HO-1 inhibition using siRNA.** (A) Representative results of RNA interference to HO-1 by western blotting. (B-C) The results of quantitative analysis of the intensity of each immunoreactive protein band. (B) HO-1 expression levels normalized to those of GAPDH. (C) SQSTM1 levels normalized to those of GAPDH. RS9 was used at 10 nM final concentration. Mean ± SEM (n = 3), ^*^P < 0.05, (Student's *t*-test). N.S.: not significant.

To further elucidate relationships between HO-1 and SQSTM1, we conducted the RNA interference experiment using siRNA against HO-1. Firstly, we checked that the efficiency of HO-1 knockdown contained the #1, #2, or #3 sequences (data not shown). Then, we chose #2 sequence which showed highest efficiency of HO-1 knockdown (Fig. 6 A-B). Next, we examined the influence of HO-1 knockdown to expression levels of SQSTM1. As a result, we confirmed the expression levels of SQSTM1 was unaltered after HO-1 knockdown (Fig. 6 A, C). The result indicates that transient induction of SQSTM1 by RS9 was independent to HO-1 pathway.

### 3.6 In zebrafish, RS9 protected retinal cells from intense light exposure *via* the autophagic pathway

**Fig. 7:**
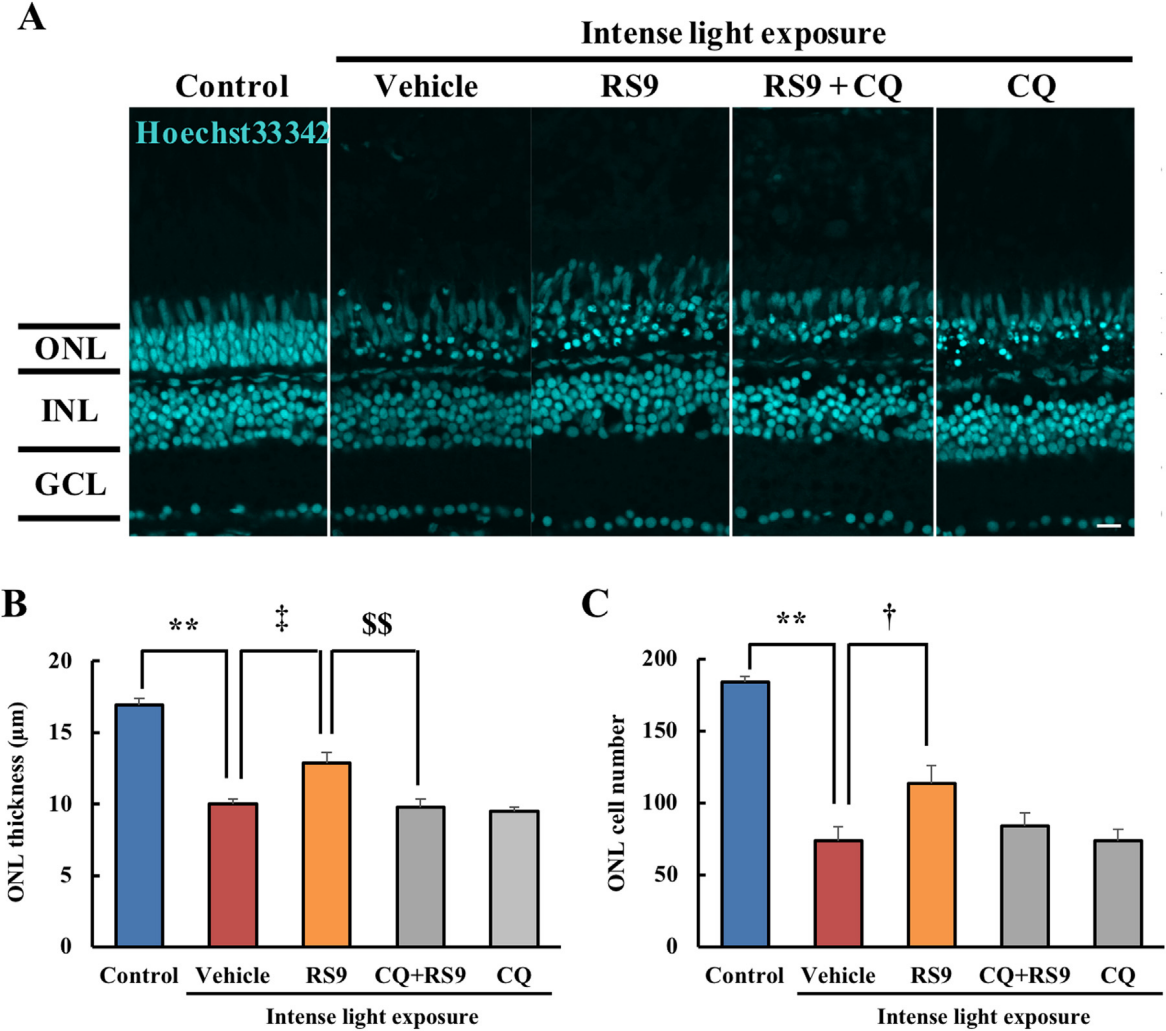
**RS9 also protected adult zebrafish retina from intense light exposure *via* the autophagic pathway.** (A-C) Hoechst33342 staining of a cryosection of zebrafish retinas injected intravitreally with RS9 and CQ. (A) Representative image (Hoechst33342: cyan). Scale bar = 10 µm. (B) ONL thickness. (C) ONL cell number. Mean ± SEM (n = 5–6). *vs*. control ^**^P < 0.01, *vs*. vehicle; ^‡^P < 0.01, *vs*. RS9; ^$$^P < 0.01. ONL: outer nuclear layer, INL: inner nuclear layer, GCL: ganglion cell layer.

Finally, we investigated whether RS9 showed same effects *in vivo*. We used the zebrafish light-induced retinal degeneration model [31]. In the adult zebrafish retina, RS9 suppressed thinning of the outer nuclear layer thickness caused by intense light exposure (Fig. 7 A-B). RS9 also prevented the decrease in the outer nuclear layer cell number (Fig. 7 A, C). Furthermore, chloroquine co-treatment abolished these protective effects of RS9 similarly to the *in vitro* experiments (Fig. 7 A-C). Taken together, the results showed that RS9 protected retinal degeneration *via* the autophagic pathway *in vivo* as well as *in vitro*.

## 4 Discussion

Autophagic degradation in RPE cells and lipofuscin deposition are closely related to each other. Cytotoxic lipofuscin accumulation is induced by the loss of autophagy function in RPE cells [4]. Healthy old retinas show abundant LC3-positive autophagosomes; however, retinas in patients with late stage AMD show a small number of LC3-positive autophagosomes [4]. These findings indicated that the dysfunction of the autophagic pathway is involved in AMD progression [4], [34]. Therefore, pharmaceutical intervention to disrupted autophagic degradation might prevent AMD progression.

The findings of the present study suggested that the Nrf2 activator RS9 could accelerate autophagic degradation and the degradation of cytotoxic abnormal proteins, resulting in cytoprotection. We observed that RS9 protected RPE cells from oxidative stress *via* the HO-1 pathway, which agreed with the results in other tissues demonstrated in previous studies (Fig. 1, Fig. 2) [16], [35]. In our experiment, co-treatment with ZnPP slightly tended to decrease the cell viability. However, the protective effect of NAC was not decreased (Fig. 2). Besides, the ZnPP concentration used in this study was less than the concentration used for HO-1 inhibition in previous studies [39], [40]. Hence, in this experiment, we considered that the influence of HO-1 inhibition strongly affected the protective effect of RS9. Notably, inhibiting the degradation of autophagosomes using chloroquine treatment also abolished the protective effects of RS9 (Fig. 3). By contrast, the protective effects of NAC were not changed by chloroquine treatment (Fig. 3). NAC is a precursor of L-cysteine that increases intracellular glutathione levels, which subsequently scavenge hydroxyl radicals [36], [37]. Therefore, we considered that the glutathione-associated redox system, which is also facilitated under Nrf2 activation [38], is not inhibited by chloroquine treatment. These results indicated another direct association of the cytoprotective effects of RS9 with autophagic degradation. Furthermore, upregulated HO-1 levels induced by RS9 were not inhibited by chloroquine treatment (Fig. 4 A-B) and cell viability under co-treatment with chloroquine and RS9 is seemed to be more weakly decreased (Fig. 3) compared with co-treatment with ZnPP and RS9 (Fig. 2). Collectively, these results suggested that the protective effects of the upregulated HO-1 levels were not inhibited by co-treatment with chloroquine and it was not responsible for all of the protective effects by RS9. In other words, these results suggest that autophagy-related protective effects certainly exist.

Consistent with the results of Fig. 3, the western blotting results showed that the relative LC3-II expression levels were upregulated by RS9 treatment (Fig. 4 A, C). Furthermore, immunostaining showed that RS9 treatment transiently increased the number of LC3-positive autophagosomes (Fig. 5 A-B). Previous reports showed that SQSTM1 was induced under Nrf2 activation [41]. In agreement with previous reports, our observation showed that SQSTM1 is transiently upregulated by RS9 treatment (Fig. 4 A, D). Similarly, immunostaining showed transient increases in the number of SQSTM1-positive speckles after RS9 treatment (Fig. 5 A, C). Based on these results, we considered that pharmacological Nrf2 activation by RS9 transiently induced SQSTM1, which accelerated autophagosome formation and abnormal protein degradation. Moreover, we also confirmed that SQSTM1 induction by RS9 was unaltered after HO-1 knockdown (Fig. 6). The result was consistent with the previous report [41].

A previous study suggested that NaIO_3_ increased the levels of cellular unfolded proteins in a concentration-dependent manner in RPE cells [26]. Moreover, some reports indicated that autophagy is associated with the unfolded protein response [28], [42]. Based on these reports, we hypothesized that NaIO_3_ most likely increases the levels of abnormal proteins, which are targets of autophagic degradation. Consistent with our hypothesis, the NaIO_3_ treatment group showed increased levels of LC3-I (Fig. 4 A, E). LC3-I is the precursor form of LC3-II. This result also indicated that NaIO_3_ treatment retarded the transition from LC3-I to LC3-II. In fact, we confirmed that chloroquine treatment alone did not induce a further decline in cell viability at 24 h after NaIO_3_ treatment (Fig. 3). Immunostaining of RPE cells without RS9 treatment also revealed vacuolated and swollen Hoechst 33342-positive nuclei at 24 h after the start of NaIO_3_ treatment (Fig. 5 A, white arrows). These features are similar to the characteristics of necrotic nuclei and a previous report demonstrated NaIO_3_-induced necrotic cell death in RPE cells [27]. These necrotic cells had diffused and high LC3 fluorescence. Based on the previous report, this diffused LC3 signal seemed to reflect the LC3-1 isoform [33]. These cells also had excessive deposition of SQSTM1-positive speckles. Taken together, these results suggested that the retardation of the autophagic degradation of abnormal cytotoxic proteins occurred at 24 h after NaIO_3_ treatment without RS9 and probably caused necrotic cell death.

Finally, we ascertained whether the protective effects of RS9 also required the autophagy pathway activity *in vivo*, in zebrafish retina. The Nrf2-Keap1 axis is evolutionarily conserved in zebrafish [43]. RS9 attenuated the damage to zebrafish retina induced by intense light exposure (Fig. 7 A-C). Previously, we confirmed that the damage to the retina caused by light exposure could be attenuated using NAC treatment [31], and that it is at least partly caused by oxidative stress. Additionally, in the present study, we found out that chloroquine treatment canceled out the protective effect of RS9 (Fig. 7 A-C). This result suggested that the Nrf2 activator produced its cytoprotective effect at least partially *via* modulating autophagy flux.

In previous report, RS9 protected ARPE-19 cells from tert-butyl hydroperoxide-induced oxidative stress and RS9 exerted maximum protective effect at 10 nM [14]. We also confirmed that RS9 exerted maximum protective effect at 10 nM against NaIO_3_-induced oxidative stress (Fig. 1). RS9 induced classical target genes of Nrf2 without oxidative stress and the effects increased concentration-dependently at 0.1–1000 nM [14]. However, the protective effects of RS9 against oxidative stress were decreased over 10 nM in spite of the cytotoxicity was not induced by 100–1000 nM [14]. Unlike these results, RS9 exerted maximum protective effects at 1000 nM against the light-induced photoreceptor cell damage [16]. As shown in the zebrafish experiment, the light-induced retinal degeneration was partially protected by RS9 treatment (Fig. 7). The light-induced cell damage is caused by not only oxidative stress but also other stresses, which like endoplasmic reticulum stress [44]. Taken together, the protective effects of RS9 against oxidative stress have optimal concentration and which is lower than against other complicated stress. We hypothesized that the reason of this is the exogeneous oxidative insult itself induces Nrf2 activation and then the proper concentration of RS9 is possibly altered. However, accurate quantification of Nrf2 activation levels is difficult. It requires more detailed analysis to confirm the hypothesis.

The determined mechanism by which RS9 causes adaptation to NaIO_3_-induced oxidative stress is illustrated in the graphical abstract. The findings of the *in vitro* experiments suggested that RS9 probably protects cells by promoting the degradation of cytotoxic abnormal proteins induced by NaIO_3_ (Fig. 3, Fig. 4, Fig. 5). Furthermore, RS9 transiently accelerates autophagy flux *via* induction of SQSTM1 (Fig. 4, Fig. 5). The pathogenesis of non-exudative AMD is still incompletely understood; there is controversy as to whether the fundamental cause of non-exudative AMD is RPE dysfunction or photoreceptor degeneration [45]. Additionally, for the turnover of the visual cycle, autophagy also contributes concertedly with phagocytosis of photoreceptor outer segments by the RPE [46]. Our *in vitro* and *in vivo* experiments showed that RS9 protects both the RPE and photoreceptor cells *via* modulating autophagic flux. Recently, growing evidence suggested that autophagy activity in the RPE is important in the progression of non-exudative AMD progression [4], [34], [47]. In conclusion, considered with these evidence, the present results indicate that an Nrf2 activator RS9 has the high potential of therapeutic efficacy to treat non-exudative AMD.
